# An Integrative Dynamic Model of Colombian Population Distribution, Based on the Maximum Entropy Principle and Matter, Energy, and Information Flow

**DOI:** 10.3390/e21121172

**Published:** 2019-11-29

**Authors:** César Cardona-Almeida, Nelson Obregón, Fausto A. Canales

**Affiliations:** 1 Department of Civil and Environmental, Universidad de la Costa, Calle 58 #55-66, Barranquilla 080002, Atlántico, Colombia; fausto.canales.v@gmail.com; 2 Director of Water Xavierian Institute, Pontifical Xavierian University, Bogotá 110311, Colombia; nobregon@javeriana.edu.co

**Keywords:** integrated modelling, social-ecological systems, maximum entropy principle, energy and information, human population distribution

## Abstract

Human society has increased its capacity to exploit natural resources thanks to new technologies, which are one of the results of information exchange in the knowledge society. Many approaches to understanding the interactions between human society and natural systems have been developed in the last decades, and some have included considerations about information. However, none of them has considered information as an active variable or flowing entity in the human–natural/social-ecological system, or, moreover, even as a driving force of their interactions. This paper explores these interactions in socio-ecological systems by briefly introducing a conceptual frame focused on the exchange of information, matter, and energy. The human population is presented as a convergence variable of these three physical entities, and a population distribution model for Colombia is developed based on the maximum entropy principle to integrate the balances of related variables as macro-state restrictions. The selected variables were electrical consumption, water demand, and higher education rates (energy, matter, and information). The final model includes statistical moments for previous population distributions. It is shown how population distribution can be predicted yearly by combining these variables, allowing future dynamics exploration. The implications of this model can contribute to bridging information sciences and sustainability studies.

## 1. Introduction

A growing human population, which also demands more energy than ever before in history, strongly depends on ecosystem services and the natural resources of the planet, whose protection and adequate management is essential for the future well-being of human societies [1]. Modern consumption habits require enormous amounts of materials from natural sources and products from industrial processes, favoring the technological flourishing of the industrial era. At the same time, the abundance and nearness of individuals have favored social benefits such as communication and learning opportunities, bringing humanity to the information revolution. The information revolution or fourth industrial revolution [2] increases the demand for both energy and materials.

Increasing research and efforts to explicitly understand human–environmental interactions have been carried out over the last decades, and there is a general agreement about the usefulness of following a systems’ perspective in the integrated analysis by modeling the management of natural resources [3,4,5]. The systems’ perspective brings us a combination of social and natural components, as well as their interactions and exchanges. The integrated analysis entails difficulties when communicating across disciplines, in the diverse data types and variables, and misperceptions about the use of social science information [6] as well as in the heterogeneity of geophysical and ecological data. To bridge the gap between social and ecological sciences research, and to foster a holistic understanding of how humans interact with the surrounding ecosystem, a number of frameworks (e.g., social-ecological systems (SES) and human-environment systems (HES)) have been developed [7] and a common approach is to consider a human subsystem and its interactions with a natural subsystem. A comprehensive review of the different methodologies and integrated analytical frameworks and classifications can be found in Binder et al. [8].

Carl Folke and Fikret Berkes coined the term social-ecological system (SES) to refer to complex, integrated systems where humans are part of nature as a whole [9,10]. SES is a useful paradigm to conduct an integrated analysis on the human–natural resources systems, offering a strong prospect toward the goal of sustainable development. It can help to address complex systems holistically by considering multiple, interacting variables and emphasizing transdisciplinarity [11]. This has shown advantages in dealing with biodiversity [12,13], water management [14,15], ecological services [16], landscapes [17], and environmental management [11], among other key topics.

There is also a wide variety of model paradigms used for representing integrated processes’ dynamics. Some of the most relevant are systems dynamics [18,19], multi-objective optimization, Bayesian networks, hybrid expert systems and feedback analysis, cellular automata, and agent-based modeling [20,21,22]. All offer different advantages and limitations according to specific structures, data required, and spatiotemporal possibilities [23]. Although there is a growing recognition that approaches integrating social and ecological knowledge should lead to more effective and sustained conservation solutions [6], there is still a need to increase the integration of these components to effectively solve environmental issues [11].

SES are thermodynamic systems, which is a way of saying they are the complex result of an intricate set of energy, matter, and information flows. However, none of these analysis frameworks or model paradigms consider information as an active flow variable closely related to all social-environmental dynamics. In previous work, Cardona [24] extended a conceptual framework treating SES as complex, resilient, dissipative, and thermodynamic systems driven by the exchanges and fluxes of the aforementioned three elemental physical entities: energy, mass, and information. Both ecological and social processes are based on energy and information, which are the basic variables of nature and life [16]. Information in SES has been treated, but still remains a concept under research. In an early conceptual work on integrated models for SES, Bellman [25] described information flows through layered systems and how it affects matter and energy exchanges through them. Ostrom [26] also pointed out some interactions among SES components based on information exchange. Information appears as the key currency for energy and matter transformations and could be the bridge to understanding the processes between social and ecological systems, and thereby changes in the environment.

Information is a notoriously multifaceted concept with different connotations in various domains [27] and its study is becoming increasingly relevant in many fields, from cellular information to social learning, and offers a new possibility to account for system changes. The paper by Kraker [28] discussed how the idea of social learning could help to improve SES management and governance, and points out how the field has advanced sufficiently to enable more rigorous and detailed empirical research. It is precisely the abundance of information in today´s society that offers, on one hand, the possibility to have a view of how information flows and affects all kind of processes, and on the other hand, allows having more tools to study, measure, understand, and use information properties and dynamics in one of our common purposes; that is, sustainable development. So, the subsequent question would be as follows: is it possible to harness this information’s relevancy for better representation and understanding of human–natural/social-ecological systems?

Within this context, this paper explores using SES dynamics to assess the relationship between matter, energy, and information flow, based on a convergent stock variable—the human population. The human population is simultaneously a result and a driver of information matter and energy dynamics. The main variable allows focusing on quantifying the effects of information on population and community dynamics, as well as on measuring field conditions [29] and exploring hypothetical relationships among SES components through energy, matter, and information dynamics.

To study the human population distribution throughout a period, we apply the MaxEnt principle to the distribution of the Colombian population. This mathematical structure enables integrating several variables as restrictions to a main variable distribution using the maximization of entropy of possible distributions. MaxEnt was introduced to ecology by Phillips et al. [30] to make estimations on the spatial distribution of species based on partial information, and it has been extensively applied in the scientific literature. On the other hand, since the work by Wilson in 1970 [31], dedicated to exploring the applications of the maximum entropy method in the field of regional planning, conceptual analogies were used to fit the statistical tool to several types of problems. From system dynamics to transportation problems and urban services locations, the usefulness of maximum entropy as a general modeling tool [32,33] is quite clear and open to a wide range of disciplines and applications.

Bajat et al. [34] analyzed human dynamics in Serbia from 1961 to 2027 using ecological niche analysis, which is not often used for human population analysis. The restrictions or predictors used were the distance to roads, elevation, slope, topographic wetness index, enhanced vegetation index, and land cover classes, which could show how human habitation preferences change in the studied time-lapse from topography features towards distance to the road network. Zhao [35] has shown the case of population dynamics in China using MaxEnt and 19 climate variables and terrain factors, which demonstrated that these were the most important ones for human settlements. Hernando and his collaborators [36] studied the population distribution in Spain based on MaxEnt principle and were able to show how macrostate distributions are connected to microstate dynamics, being able to replicate behaviors such as migrations and big cities’ saturation. The most relevant aim of that work is to demonstrate the applicability of MaxEnt to collective human behavior, and the potential use of an explicit social thermodynamics.

This paper proposes a first approach to a socio-ecological system (SES) model based on a conceptual framework constituted by the combination of energy, matter, and information dynamics. The novelty of the proposal lies in the hypothesis that the information dynamics can be considered a central variable in the model, and simultaneously assessed along with matter and energy variables in an approach that would allow describing the distribution of the human population. This approach of considering information as a main variable in models of SES has been previously discussed by Bellman, Ostrom, and Jorgensen, among others, but attempts to use it usually involve it as an external data source or signal. The model presented in this paper is the first one, according to the knowledge offered, that addresses this issue based on easily available datasets for most regions of the world.

This paper presents a brief discussion of the conceptual framework as the basis of the model’s construction. It continues with statements on the population as a central variable and the identification of complementary variables related to information, energy, and matter, as well as the inclusion of statistical moments for the mathematical model construction. Subsequently, the model’s results are analyzed regarding its performance and suitability.

## 2. Methods

Here, we present a brief conceptual discussion to explain how matter and energy were analyzed, and how to relate to information, which is analyzed more in-depth to propose possible manners to evaluate and integrate it into a model.

Later in the title, the model’s construction details are presented. The model must fulfill the purpose of integrating variables related to information, matter, and energy, and being applied to a social-ecological system, this is, considering the human population. The following considerations summarize the model requirements:

- The human population must have a central role, consistent with the idea of exploring social-ecological systems.
- Given the complexity and extent of fluxes and exchanges in the systems, indicator variables could be eligible to test the global idea to start building the relational hypothesis thereafter.
- The system must be defined clearly, so a territorial unit was chosen.
- The data of selected variables must be available homogeneously in the chosen system/territory extension, and throughout the time.
- Given the complexity of the system, the model could be built based on statistical or heuristic structures, not necessarily using functional structures.

### 2.1. Energy and Matter

As a legacy from ecology and economy, the SES has a wide range of models and paradigms accounting for energy and matter flow and balances among the system’s components. Georgescu-Roegen, in the early seventies, pioneered a theory on entropy and the economic process [37]; though it was widely debated later, it is considered a pillar for establishing a converging point between ecology and economics [38]. In ecology, from Lotka [39] and Lindeman [40], the notion of energy and nutrients flows in food chains is a study field.

Several frameworks have approached matter and energy balance in SES. MEFA (matter and energy flow analysis) has been widely applied [41,42]. Suh [43] offered a generalized framework to incorporate matter and energy flows in an integrated ecological and economic system. Liao and collaborators [44] proposed a framework based on an analysis of the thermodynamics of Industrial Ecology, which is the interaction subsystem between human and ecological systems. Gianpietro [45,46] presented the multi-scale integrated analysis of the societal and ecosystem metabolism (MuSIASEM), more oriented to processes and scales. The paper by Gerber and Scheidel [47] reviewed the two aforementioned methodologies to assess their strength and limitations in analyzing the metabolism of social systems.

Through his ecological law of thermodynamics (ELT) [48,49,50], Jorgensen has argued how ecosystems’ fundamental processes are based on matter, energy, and information flow. According to ELT, from a set of possible configurations, an ecological system will adopt the one that is the farthest from thermodynamic equilibrium. That means a configuration that offers the most intricate and efficient use of low entropy energy. To fulfill that law, an ecological system can develop by increasing biomass, extending and reinforcing energy dissipation networks, and increasing complexity and organization.

Cardona [24] proposed a simple balance useful to visualize and relate matter and energy processes with information in a SES. Matter stock varies depending on internal processes, source–sink reactions, and external exchanges with other systems. For two different times, the variation in the amount of matter can be expressed as follows:

(1) $$m_{t1}-m_{t0}=m_{input}-m_{output}+m_{processes}+m_{sources}.$$

In the same system, energy exchanges are represented by low-entropy energy (useful) entering the system and energy fixed by the system (e.g., photosynthesis), both of which add to the increase of internal energy, while energy diminishes simultaneously by the work exerted and low-entropy energy dissipated. Over a period, an expression to represent system energy is as follows:

(2) $$e_{t1}-e_{t0}=e_{useful}+e_{fixed}-e_{work}-e_{dissipated}.$$

In Equations (1) and (2), the transformations represent an infinite number of configurations of substances, states, and energy forms, and a complete balance can become impossible. However, it illustrates how each term depends on the interactions and transformations of energy, matter, and information. Here, we present a simple distinction; that is, a spontaneous transformation results from available energy and accessible substances, no information is used for the transformation, even when it can be generated in the event. A conditioned transformation, on the contrary, will create changes in matter balance using available energy and useful information. This implies that an information user is an organism or organization that is only capable of profiting from energy or matter by means of information use. A wildfire, for instance, produced by a lightning is a spontaneous transformation, but a dam built across a river is a conditioned transformation.

As SES are constituted basically by organisms and organizations, whether social or ecological, the SES development is based on conditioned transformations and, consequently, on information.

### 2.2. Information

From Boltzmann to Landauer, information has gained its place as a real physical entity [51], but from Schrodinger’s reflections on life and information, the debate has been open and the meaning of the information concept is further than the physics or mathematics realm.

Vihervaara et al. [16] offer a clear and concise review of information concepts in different contexts, from physics to biology, including the mathematical concept from Shannon. Jorgensen [50] establishes a positive correlation between complexity, energy flows, information content, biodiversity, and redundancy of the ecological system, and Vihervaara uses this as a basis to asses environmental services based on information content and energy, the concept of embodied energy from Odum [52].

Ecology and biology studies have addressed issues such as the importance of information in evolution theory [53], based on organisms’ capacity to record environmental conditions in their genetic code [54,55]; have discussed the effects of social information on individual behavior in animals [29]; and have modeled human–wildlife interactions based on information exchanges [6], as well as the relationships between information transmission and social structures [56], among others.

Dolgonosov presented a parallel between information and the human population and represents its dynamics through history [57] and into the future [58] by defining knowledge functions as the information recognition capacity. Lange [59] studies the structure of information flow in groups using social network analysis, demonstrating how it is possible to incorporate these approaches into conservation intervention strategies. Siebenhüner and others [60] show a review regarding social learning in the context of ecological economics and reveal how the increase of published research has still not yet delineated a dominant conceptual approach for the study of the subject.

#### Information Balance in SES

The idea of information flow or transformation in an SES to perform a sort of balance and establish it as a central variable involves several considerations explained here. In socio-ecological systems, information is exchanged with the surroundings through the system’s boundaries, while also flowing in internal processes and subsystems. Information can be transferred, but the increase inside the systems is not easily linked to gross inputs and outputs as matter or energy. Still, it is reasonable to think that the input of information contributes to the increase or change of the previous amount of information in the system. It does not happen the other way around, though. The information delivered or sent to the surroundings does not necessarily reduce the content of information inside the system. Information can be replicated, transferred, or destroyed [55] by causing energy cost, but the information also could be created, in the sense of order, inside the organism or organization, with the cost being their own energy [61,62]. That means that the increase in the total content of information in the system will cost energy and, reciprocally, more energy dissipation will demand more information [48,49,63].

Here, we state two basic ideas discussed above: (a) more energy allows more information production and more information (organization) allows more efficient energy dissipation, (b) raw information content is positively related to more refined information (organization, knowledge, communication, and learning).

With that in mind and considering the final remark in Section 2.1, we can expect the content of information to be related to organization, knowledge, and learning, which favors conditioned transformations, which are required for SES development. Shannon’s probabilistic approach accounts for information in the sense of uncertainty [64] associated with a random variable, but it lacks quantifying the information’s meaning. Zurek, in contrast, assures that information cannot be separated from its meaning [65]. Assessing the content of information in a system is a complex task [66,67]. Estimations of the global information have been carried out by quantifying information contained in text pages of all books ever written [57,68] or supposing all the information has been digitalized [69].

According to Dolgonosov [57], each organism contributes to an amount of finite information through its genetic code, its neural capacity, and currently an amount of information in external media $i_{system}=i_{genetic}+i_{neural}+i_{ext-media}$. The estimates of information are not concerned with information exchange and transmission.

Living organisms are characterized by their ability to perceive and analyze information about their environment [54] and they are, at the same time, information users and encoders [54,57], with the purpose of obtaining and using useful energy; in the words of Boltzmann, life is the struggle for free energy [48]. An individual will prioritize processes where it can find information useful to get energy. These processes are information exchange, communication, cooperation reproduction, and so on. According to this, the conceptual model by Cardona [24] states that a system’s information depends not only on the number of organisms, but on their informational interactions.

The individuals or organizations in the system use and enrich the content of information through their interactions and information exchanges. For all of the above, the system’s information would be as follows:

(3) $$i_{system}=i_{individuals}+i_{external\;media}+i_{exchange},$$

where $i_{individuals}=i_{genetic}+i_{neural}$. It has been argued how grosser information favors more structuring and complexity in it. While this does not address the distinction between information and knowledge, it is expected that measuring any information variable provides a better understanding of learning and the system’s organization capability. In human communities, for instance, governmental and educational establishments boost the flows of information to individuals and other organizations, enabling the exceptional development of societies.

### 2.3. Construction of the Model

Any living organism could be considered as a special combination, convergence, or specific arrangement of information, matter, and energy flows. Human society will be a greater and even more complex combination of these factors. In the proposed model, the energy, matter, and information accounts can be described by population. At the national level, throughout a period, population is related to aggregated variables representing the macro-scale or macro-state of the “nation” system. Similarly, the population distributed in space in regions or provinces will affect matter, energy, and information processes at the regional scale. This is called the system’s microstates and its change in space and time will depend on specific regional sets of patterns of energy, matter, or information production/consumption rates.

These sorts of relationships at the macro- and micro-states come close to the idea of distribution analysis using the maximum entropy principle proposed by Edwin Thompson Jaynes [70]. A complete description is presented by Levine and Tribus [71]. According to the principle, the allocation of probabilities to an unknown distribution is that which maximizes the entropy subject to the information available [72]. In the case of a population, these restrictions to the maximization can be understood as systems’ macro-state conditions and their regional distribution. The restrictions are configured to involve information, matter, and energy flows.

#### Mathematical Structure

If $p_{i}$ is the probability of a given variable X:{x_i_}, the function to maximize is Shannon’s entropy: $H\;\left(P\right)=-\sum_{i=1}^{N}p_{i}\log_{e}p_{i}$ [73]. The univariate, discrete, and general form of the principle is given by the following:

(4) $$\begin{array}{l} H\left(P\right)=-\sum_{i=1}^{N}p_{i}\log_{e}p_{i}=-\sum_{i=1}^{N}p_{i}\ln p_{i},\\ \mathrm{Subject}\;\mathrm{to}:\sum_{i}^{N}p_{i}=1,p_{i}\geq 0,\;i=1,2,\ldots ,N,\\ \sum_{i=1}^{N}p_{i}g_{\mathrm{ri}}=C_{r},\;r=1,2,\ldots ,m, \end{array}$$

where $g_{\mathrm{ri}}=g_{r}\left(x_{i}\right)$ it is the *R*-th function of X: {x_i_} to express the restriction C_r_ and m is the number of restrictions. Mohammad-Djafari [74] developed a computational algorithm generalized for continuous variables. This algorithm was adapted and used as a calculation engine in the model. 

Departments are territorial units (TUs) comparable to the concept of province or state. Let the national population be $B_{t}$ for any year t, and $b_{i,t}$ the population in the i^th^ department in the year t. Thus, the following proportion:

(5) $$p_{i}=\frac{b_{i}}{B_{t}},$$

represents the distribution of the population in departments or TUs. The natural restriction is $\sum_{i}^{n}p_{i}=1$.

Several modeling exercises were carried out, first considering the natural restriction, the number of students (information), electrical consumption (energy), and water demand (matter) as restrictions called physical or flow variable restrictions. Furthermore, restrictions given by statistical moments of the population’s distribution were included to involve social trends and previous states of the distribution. Each variable and the restriction in the form $\sum_{i=1}^{N}p_{i}g_{\mathrm{ri}}=C_{r}$ is presented next.

### 2.4. An Informational Variable

We have argued that a larger population favors more information production, given the number of individuals and some specific patterns of consuming/producing information. Also, more information is related with complexity/organization and that is associated with social institutions. Avoiding the discussion on the quality of the information, it is assumed that institutions and their original products denote social organization and a more complex system.

To define an informational variable, several concepts and available data were considered. Two kind of variables were explored, products of information exchange and organizations themselves. In the first case, the focus was on technical or scientific production, artistic products, and legislation or normativity. In the second case, the focus was placed on the number of educational, governmental, or productive institutions. The data explored included national legislative production, number and size of governmental institutions, number and size of industrial organizations, registry of artistic production, and national editorial industry, among others, but none of these data were as comprehensive and homogeneously registered as those from the educational sector and scientific production.

Scientific knowledge or new knowledge attained by social arrangement can be compared, for example, with the number of scientific papers produced in different fields of knowledge. The number of publications summarizes the collective effort to generate new knowledge and represents a well-organized and well-structured system, as well as research-oriented educational institutions. It also requires financial and governmental support, policies, and facilities, as well as abundant students, to support the synthesis of information in a sort of pyramidal structure. However, scientific production is a dataset available at the national level; no information was found at the TU scale. On the basis of the idea of a pyramidal structure for scientific production, and also on the analyses presented by Cardona [24], where he shows how scientific production in Colombia is linearly correlated with the number of universities, the number of research groups, and the number of students, this was selected as an informational equivalent or indicator variable.

**Figure 1:**
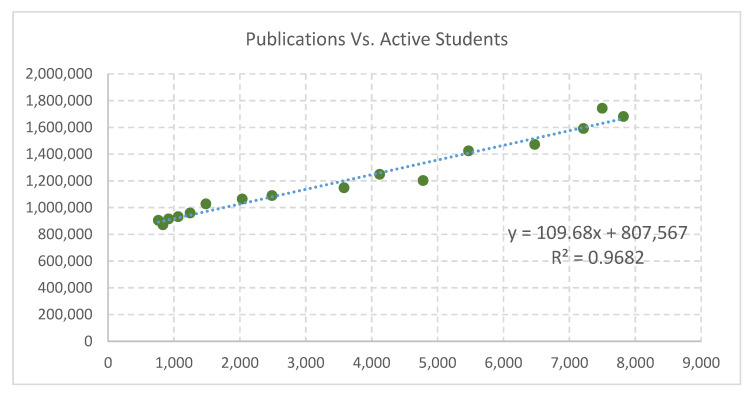
Scientific publications versus the Number of active students in higher education. Horizontal axis: total of publications; vertical axis: number of students. Data are aggregated yearly.

Using a record of the number of scientific papers published per year by Colombian researchers from 1995 to 2015 from SCImago National Ranks-Jrs [75], and compared to active students at Universities and Technical Educational Institutions, a linear correlation is observed (see Figure 1). Data on students enrolled in higher education are available from the Ministry of National Education’s Information System, MEN-SNIES.

#### Educational Rate

In a TU, the relationship between population $b_{i}$ and the number of students $u_{i}$ is $\alpha_{i}=\frac{u_{i}}{b_{i}}$, the higher education or training rate. To build a restriction on the synthesis, production, or availability of information, it can be expressed as $b_{i}.\alpha_{i}=u_{i}$,

(6) $$\sum_{i}^{n}b_{i}.\alpha_{i}=U_{t}.$$

The summation of all TUs of the amount of inhabitants times the local training rate equals the total number of students in the country $U_{t}$, which is a system macrostate.

The distribution of students is non-uniform; on the contrary, the presence of students in a TU will be likely according to regional conditions. In a similar way to $p_{i}$,

(7) $$q_{i}=\frac{u_{i}}{U_{t}},$$

where $q_{i}$ corresponds to the weighting factor in each TU and according to the definition of the mean, it corresponds to the probability of $u_{i}$. As a result, an average of students per department should respond to the following:

(8) $$\sum_{i}^{n}q_{i}.u_{i}=\bar{u_{t}}.$$

Replacing, conveniently, $\sum_{i}^{n}q_{i}.b_{i}.\alpha_{i}=\bar{u_{t}}$ and $\sum_{i}^{n}q_{i}.(p_{i}.B_{t}).\alpha_{i}=\bar{u_{t}}$. Including now the definition of $q_{i}$,

$$\sum_{i}^{n}\frac{u_{i}}{U_{t}}.(p_{i}.B_{t}).\alpha_{i}=\bar{u_{t}}\;\mathrm{and}\;\sum_{i}^{n}\frac{B_{t}}{U_{t}}.p_{i}.u_{i}.\alpha_{i}=\bar{u_{t}},$$

if $A_{t}=\frac{U_{t}}{B_{t}}$, represents the national average university education rate, and thus the following is obtained:

(9) $$\sum_{i}^{n}p_{i}.u_{i}.\alpha_{i}=\bar{u_{t}}.A_{t}.$$

### 2.5. Energy Variable

In the case of the energy variable, fuel for transportation, oil exploitation, electricity consumption, and primary productivity were considered among others. Electrical consumption is selected as the energy-related variable given the wider availability of data and its relation to information dynamics given the rising use of informatics and computational technologies. In relation to water, hydropower generation in Colombia is of major importance, and thus possible interactions can be further depicted. Total consumption $ce_{i}$ in a TU is estimated from the following expression:

(10) $$ce_{i}=\epsilon_{i}*b_{i},$$

where $\epsilon_{i}$ is the factor or module of electricity consumption per capita in that particular TU, similarly for the development for the Equation (6), the following can be argued:

$$\sum_{i=1}^{n}b_{i}.\epsilon_{i}=\sum_{i=1}^{n}ce_{i}={\mathrm{CE}}_{t},$$

where ${\mathrm{CE}}_{t}$ is the national electrical consumption in year *t*. The electrical weighting factor is $qe_{i}=\frac{ce_{i}}{{\mathrm{CE}}_{t}}$; therefore, $\sum_{i=1}^{n}qe_{i}.ce_{i}=\bar{ce_{t}}$ and $\sum_{i=1}^{n}\frac{ce_{i}}{{\mathrm{CE}}_{t}}.p_{i}.B_{t}.\epsilon_{i}=\bar{ce_{t}}$.

Considering $E_{t}=\frac{{\mathrm{CE}}_{t}}{B_{t}}$,

(11) $$\sum_{i=1}^{n}p_{i}.ce_{i}.\epsilon_{i}=\bar{ce_{t}}.E_{t},$$

where $E_{t}$ is the national average consumption module and $\bar{ce_{t}}$ is the average departmental consumption for the year *t.*

### 2.6. Matter Variable

In the case of matter, the variable selected was water demand from options such as agricultural products, industrial raw materials, and international trades. As in the case of electrical energy, data about water are available and rather abundant. Also, the fact that water is related to diverse human activities and environmental processes is important, thus allowing to preview further cause–effect relations or models. The water demand per capita in a TU is $w_{i}$, where total demand will be the following:

(12) $$cw_{i}=w_{i}*b_{i}.$$

In the homologous form, now for the case of water demand, the following is obtained:

$$\begin{array}{l} \sum_{i=1}^{n}cw_{i}={\mathrm{CW}}_{t},\;\mathrm{considering}\;qw_{i}=\frac{cw_{i}}{{\mathrm{CW}}_{t}},\\ \sum_{i=1}^{n}qw_{i}.cw_{i}=\bar{cw_{t}},\\ W_{t}=\frac{{\mathrm{CW}}_{t}}{B_{t}}, \end{array}$$

(13) $$\sum_{i=1}^{n}p_{i}.cw_{i}.w_{i}=\bar{cw_{t}}.W_{t},$$

where ${\mathrm{CW}}_{t}$ is the total national consumption for the year *t*, $W_{t}$ it is the national average per capita consumption module, and $\bar{cw_{t}}$ the average departmental consumption for the year *t*.

Table 1 summarizes all variables expressions and abbreviations for a better understanding of the model. 

### 2.7. Statistics as Initial Conditions

In order to include information about previous microstates of the system, to find the dynamic evolution of the model, several restrictions based on statistical moments were included. The mean and standard deviation, for instance, from the previous period can improve the results of population distribution. Conceptually, that can be conceived as a tendency of the population to maintain its current distribution given social behaviors such as the affective attachment to a place, family, or cultural bonds, among others. On the basis of Equation (1), for an *r* number of restrictions, and with $g{\left(x_{i}\right)}_{r}$ being the index i, the restrictions can be set as follows:

(14) $$\sum_{I=1}^{N}i^{m}p_{i}=C_{m},$$

where $C_{m}$ is the mth statistical moment estimated from currents p_i_ available [76].

## 3. Results from the Model

The full expression of the MaxEnt integrated model for the restrictions of the three flow variables and *m* constraints of statistical moments is presented in Equation (15).

**Figure 2:**
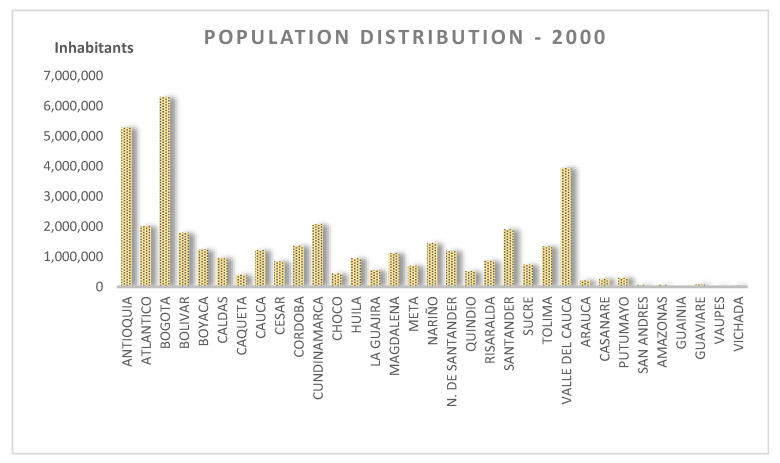
Population by department/province in Colombia for the year 2002 (Source: DANE—National Administrative Department of Statistics).

Official information was used in the model as reported by National Administrative Department of Statistics (DANE, by its acronym in Spanish) based on the 1985, 1993, and 2005 censuses and projections to 2020. The total number of departments and the district of Bogotá add up to 33 departments or TUs; Figure 2, shows the number of inhabitants by department in 2002 arranged according to a departmental code, an official standardization used by the DANE.

Data corresponding to the average water demand in 2007 were taken from Public Services Information Services in Colombia (SUI, by its acronym in Spanish). Electrical consumption values were taken from yearly reports by the Mining and Energy Planning Unit (UPME, by its acronym in Spanish), the national agency in charge. Restriction variables are presented in number of students, total electrical consumption in KW h, and total water demand in m^3^. In addition, the production or consumption rates must also be known: $\alpha_{i}$, $\epsilon_{i}$, and $w_{i}$.

### 3.1. Calibration and Verification

The calibration process consisted of modifying, including, or combining restrictions into the MaxEnt structure, while searching for the best fitness in the yearly population distribution. Combinations considered the natural restriction, the three restrictions to flow variables, and from 2 to 20 statistical moments and data indexing in descending or bell-shaped order. Two sets of experiments are presented here. One exploring the number of statistical moments to consider and the second combining physical variable restrictions.
$\max\left(H\left(\mathrm{pi}\right)=-\sum_{i=1}^{N}p_{i}\ln p_{i}\right);\;\mathrm{Subject}\;\mathrm{to}:$(15)r_0._ Natural restriction$\sum_{i}^{n}p_{i}=1$r_1._ First restriction For 33 departments and macro-state: national rate of training per student average.$\sum_{i}^{n}p_{i}.u_{i}.\alpha_{i}=\bar{u_{t}}.A_{t}$ $\sum_{i=1}^{n}p_{i}.u_{i}.\alpha_{i}=\left[p_{1}\cdot \alpha_{1}\cdot u_{1}+p_{2}\cdot \alpha_{2}\cdot u_{2}+\cdots +p_{33}\cdot \alpha_{33}\cdot u_{33}\right]=\bar{u_{t}}.A_{t}$r_2._ Second restriction For the case of n = 33 departments and the national macro-state of electrical consumption$\sum_{i=1}^{n}p_{i}.ce_{i}.\epsilon_{i}=\bar{ce_{t}}.E_{t}$ $\sum_{i=1}^{n}p_{i}.ce_{i}.\epsilon_{i}=\left[p_{1}\cdot ce_{1}.\epsilon_{1}+p_{2}\cdot ce_{2}.\epsilon_{2}+\cdots +p_{33}\cdot ce_{33}.\epsilon_{33}\right]=\bar{ce_{t}}.E_{t}$r_3._ Third restriction For the case of n = 33 departments and the national macro-state of water consumption$\sum_{i=1}^{n}p_{i}.cw_{i}.w_{i}=\bar{cw_{t}}.W_{t}$ $\sum_{i=1}^{n}p_{i}.cw_{i}.w_{i}=\left[p_{1}\cdot w_{1}\cdot cw_{1}+p_{2}\cdot w_{2}\cdot cw_{2}+\cdots +p_{33}\cdot w_{33}\cdot cw_{33}\right]=\bar{cw_{t}}.W_{t}$r_4._ Fourth restriction For the case of moments with ascending indexes.$\sum_{i=1}^{n}p_{i}g_{\mathrm{ri}}=\left[p_{1}g_{1,1}+p_{2}g_{1,2}+\cdots +p_{33}g_{1,33}\right]=C_{1}$ $\sum_{i=1}^{n}p_{i}g_{\mathrm{ri}}=\left[p_{1}\cdot 1+p_{2}\cdot 2+\cdots +p_{33}\cdot 33\right]=C_{1}$⋯r_m_.m ^th^ restriction For the case of moments not centered with ascending indexes.$\sum_{i=1}^{n}p_{i}g_{\mathrm{ri}}=\left[p_{1}g_{m,1}+p_{2}g_{m,2}+\cdots +p_{33}g_{m,33}\right]=C_{m}$ $\sum_{i=1}^{n}p_{i}g_{\mathrm{ri}}=\left[p_{1}\cdot 1^{m}+p_{2}\cdot 2^{m}+\cdots +p_{33}\cdot 33^{m}\right]=C_{m}$

Calibration was carried out with data between the years 2000 and 2010. Subsequently, a verification or checking of the model was performed with data between 2011 and 2015.

**Figure 3:**
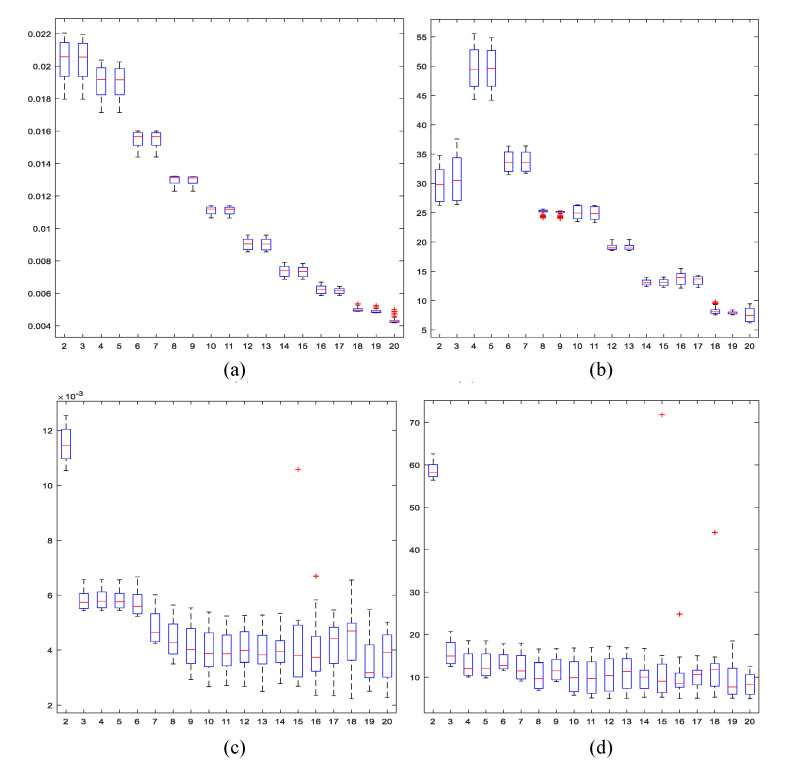
*p_i_* estimation error. Twenty statistical moments (horizontal axis). Root Mean Square Error - RMSE from the model with data indexed in centered bell-shaped order (**a**) and relative error (%) (**b**). For data organized in descending order, RMSE (**c**) and relative error (**d**).

Initially, the statistical moments did not match the population distribution with the data arranged according to the original codes shown in Figure 2. Tests with data distributed in bell-shaped order, disposing of more populated TUs in the middle, as well as in descending order, were evaluated. Illustrating results are presented in Figure 3, where it is shown how the error diminishes consistently only with the data-indexing organization. Experiments using non-centered moments and a descending order of data show greater and faster adjustment, achieving average relative error values of less than 10% from the eigth moment.

#### Physical Variable Restrictions

**Figure 4:**
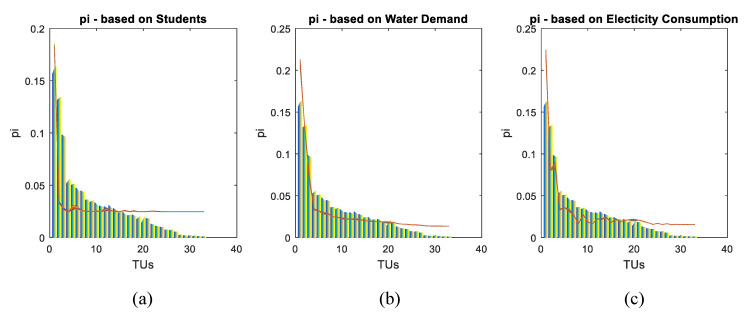
Marginal models. p_i_ estimations based on natural restriction and (**a**) number of students, (**b**) water demand, and (**c**) electricity consumption. territorial unit’s (TU’s) index from 1 to 33 on the horizontal axis. Values of $p_{i}$ on the vertical axis. Real $p_{i}$ in colored bars and estimated $p_{i}$ in colored lines.

Each of the discussed physical variables and the natural restriction were separately evaluated in order to have an overview of their capability to represent the system’s state, but the results were unsuccessful. Figure 4 illustrates the results of these marginal evaluations. In all graphs, the actual value of each variable is presented in the color bars for the 33 TUs and varying tone from year 2000 (dark blue) to 2010 (yellow). The colored lines, different for each year, link points of the estimated values of the population distribution $p_{i}$.

**Figure 5:**
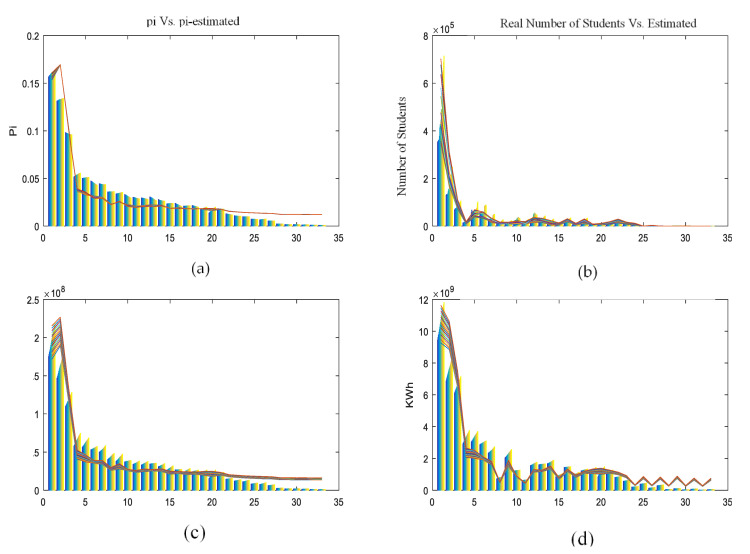
Integrated model. Real values in colored bars and estimated in colored lines. Results of estimated *p_i_* show a big deviation from real *p_i_* values, especially in lower populated TUs (**a**). Estimation of number of students (**b**), water demand in cubic meters (**c**), and electrical consumption in kW/h (**d**). All the estimates are based in modeled *p_i_* values from the integrated first model.

Variables were combined in the following cases: water–energy, water–number of students, and energy–number of students. However, the best result was obtained from combining the three of them, as shown in Figure 5a.

Figure 5 also shows, as a form of control, estimated values of student population (top right), water demand (bottom left), and electrical consumption by TU using modeled values of $p_{i}$.

Fairly acceptable goodness-of-fit is observed between the estimated data and actual values. Acceptable in terms of relation, causality, and trends. However, the estimation of relative errors is not favorable for the possible applications of the model. Errors were particularly considerable when assessing low populated territories, which are usually associated with poor quality of life. Departments 1 to 25 show relative estimation errors for $p_{i}$ around 15%, while the groups from 26 to 33 show departments with very few populations, with errors exceeding 300%. These results led to the consideration of variables representing previous states of the population distribution, that is, statistical moments.

### 3.2. Integrated Model

**Figure 6:**
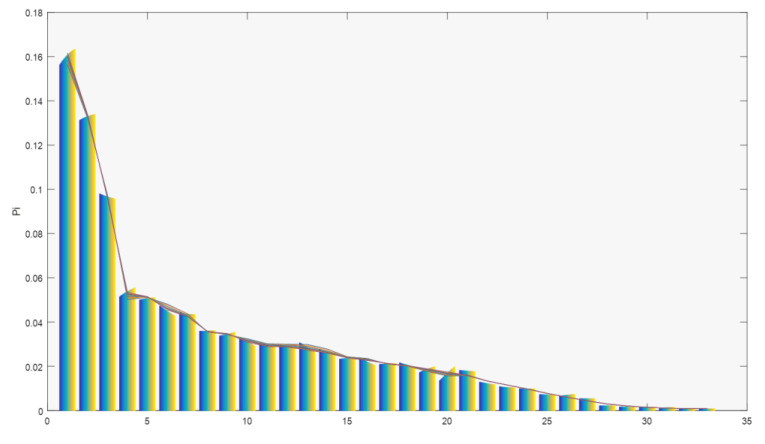
Estimated *p_i_* (colored lines) versus real *p_i_* (colored bars). Horizontal axis: 33 departments in descending order.

The results for the optimum model, combining three flow variables and ten statistical moments, with data arranged in descending order, are presented in Figure 6 where the real and estimated *p*_i_ are shown.

**Figure 7:**
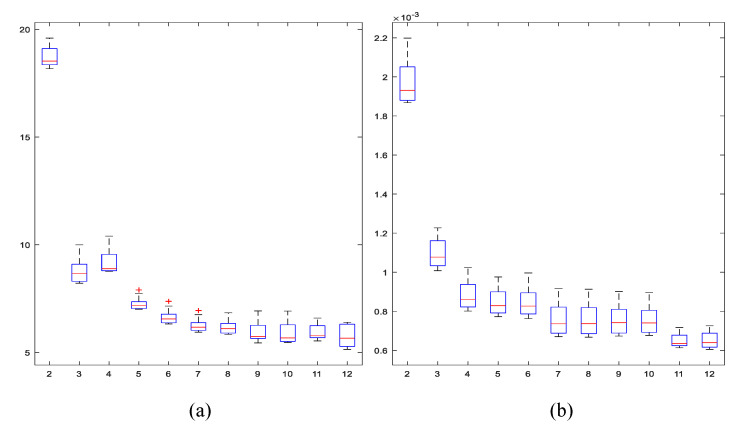
Relative error in percentage (**a**) and RMSE (**b**) for the integrated model and up to 12 moments (horizontal axis).

Errors found with this complete model are synthesized in Figure 7. The relative error with the current integrated model reaches 5.9% with 10 statistical moments, but shows an error below 10% even from the third statistical moment.

**Figure 8:**
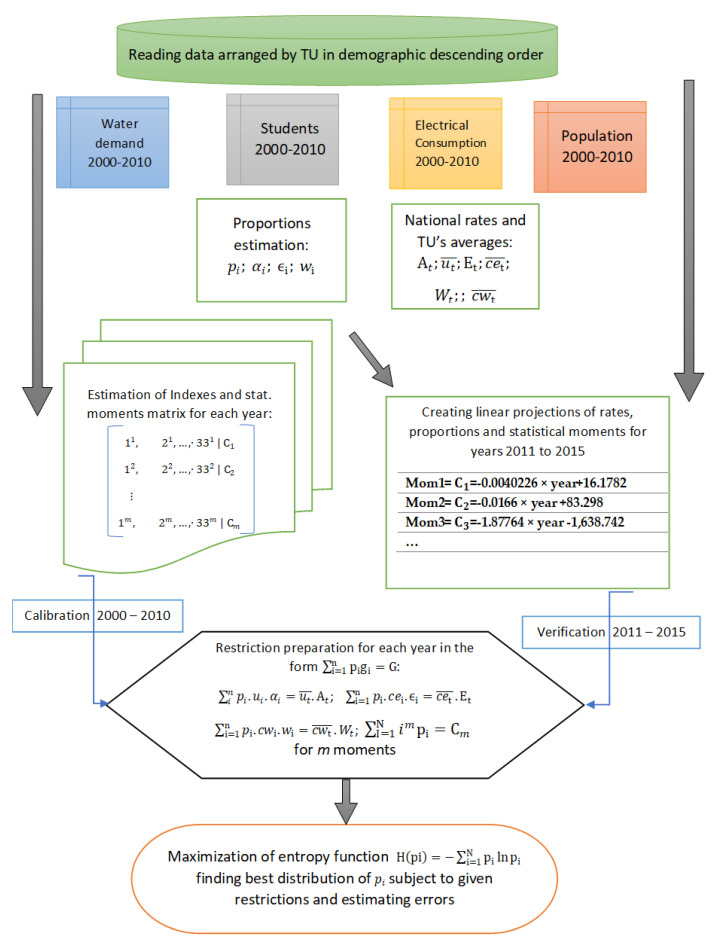
Model algorithm representation.

In the verification process between 2011 and 2015, coefficients or parameters were unknown in each time step. Production rates related to water, energy, and information per capita were considered to be constant, but the net production per department was estimated based on linear regressions. Table 2 gives the linear regression parameters in an equation of the form *y* = *mx* + *b*, for each statistical moment, where *x* is a given year. Figure 8 presents a graphical summary of the model algorithm, which also illustrates the difference between the calibration and verification processes.

**Figure 9:**
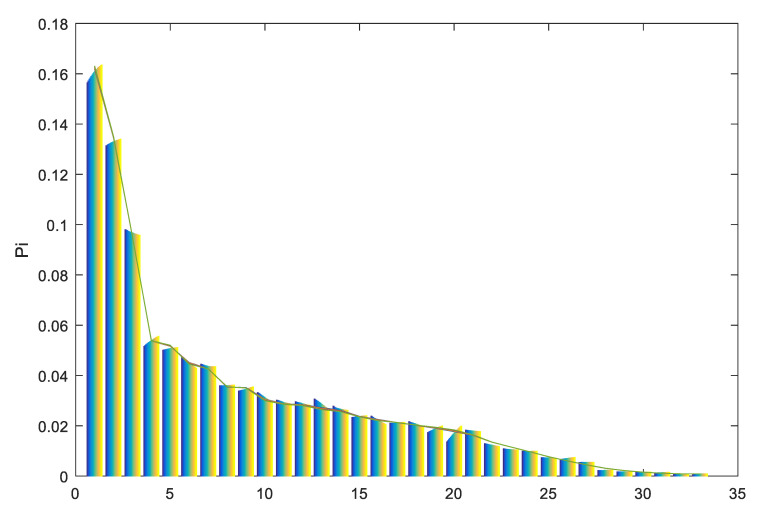
Estimated *p_i_* versus real *p_i_* (bars), between 2011 and 2015. TU’s index from 1 to 33 on the horizontal axis. Values of $p_{i}$ on the vertical axis. Real $p_{i}$ in colored bars and estimated $p_{i}$ in colored lines.

Figure 9 presents the results for the period between 2011 and 2015, where acceptable goodness-of-fit is observed. The average relative error value was 6.55%, which is quite close to the calibration relative error of 5.95%.

The mean quadratic error was below 0.0008, which gives a reasonable idea of the model error. Observing Figure 9, a good fit in estimated data is evident. However, for TU with a very small population, the model showed less precision in the estimation and errors were relatively larger compared with more populated TUs. In the case of Bogotá (*p_i_* = 0.16), the Root Square Mean Error - RMSE implies an uncertainty associated population between 7,196,400 and 7,203,600 inhabitants. That is a variation of 3600 inhabitants or ± 0.05%. In the same exercise for Caquetá, a low populated TU (*p_i_* = 0.01), the variation in the number of inhabitants would be ± 0.8%.

## 4. Discussion

Aiming to explore the hypothesis of finding an informational variable and relating it with matter and energy, we proposed a new model choosing population distribution as the central variable. That was motivated by the idea of profiting from the “information” availability to contribute to SES dynamics’ modeling and understanding. Variables related to energy, matter, and information, called physical variables, were identified and integrated under the structure of MaxEnt, to represent the human population distribution in Colombia. The specific variables selected were students in higher education, water demand, and energy consumption, and statistical moments were also included to involve previous states of population distribution.

The goal of identification of an informational variable was successful and it was effectively integrated into a model with the other variables. For the informational variable, a linear relation can be observed between technical/scientific information production and the quantity of higher education students in Colombia. This is a valuable finding that can lead to a wider description of relations among scientific products, amount of students and population, and in general to information dynamics in human systems and SES. The proposed model was able to reproduce the human population distribution and its dynamics in Colombian regions. However, the incidence of the physical variables over the population distribution was not predominant and statistical moments of the population data itself were included in the model.

The first exploration of variables’ combinations was carried out with physical variables, but a significant error on the estimations of population distributions was found, especially in low-populated regions. To explain this behavior, a conjecture was that physical selected variables describe well-organized societies with access to public services and education. Precisely, low-populated regions in Colombia have a lack of these conditions and, in consequence, the population is not determined by access to the selected variables. The inclination to reside in low-populated regions can be attributed, among other causes, to social bonds and traditions as driving forces. In consequence, restrictions related to the inner population state in a previous time aimed to represent lifestyle traditions were included in the form of statistical moments, obtaining the improved performance described in the model.

Population dynamics showed a causal relation with each defined variable, but as mentioned, the marginal results were not satisfactory. On its own, the model exclusively with statistical moments was able to reproduce population distribution with ten moments and a relative error of 10%. The integrated model with physical and statistical restrictions was able to improve relative errors up to 5.9%, and the results suggest some degree of consistency, enough to not reject the hypothesis of an informational variable capable to add in an integrated model. From a technical perspective, statistical moments can be considered as an informational variable. However, this is extracted from the central variable, and the expectation was to understand and explore information as a physical entity interacting in the system.

There is no extended research exploring how to address information as the main variable on SES analysis. Dolgonosov, however, estimated a global human population dynamic model, based on information content [57]; we proposed a model of population distribution in a single country, and unlike Dolgonosov, the informational variable did not depend exclusively on the information content contributed by each individual; in our case, the informational variable arises and is enriched by the interactions of individuals.

Vihervaara et al. [16] remark on the importance of information content in biodiversity and its relationship with energy content (biomass). They suggest a manner to instrumentalize the information accounting by biodiversity under the ecosystem service approach. In parallel, the conceptual framework we used to build the model has the premise that richer informational exchange will be surrounded by greater energy and matter transformation and flows in an SES. The model results reinforce that hypothesis when cities and regions with more intense information dynamics, represented by students and institutions, attract more population and increase water and energy exchanges. However, more studies implementing either this model in different countries/contexts or different measures/indicators of social informatic interactions in the current model are necessary. 

It was possible to adapt the MaxEnt principle and methodology, to perform a dynamic population distribution model. The model results showed a performance useful to explore variables or relevant aspects in regional or national management in a time span from five to ten years. Analysis of population distribution scenarios could be made for resource management and regional planning; for example, in the case of regional transport, population distribution and growth, demand for services, health, education, or market studies.

MaxEnt is an extended statistical tool, but is scarcely used to study human population dynamics. Hernando et al. [36] showed a model in which MaxEnt was used to study the human population in Spanish municipalities. Different from our work, they considered Q-growth functions as restrictions inside the MaxEnt structure. Their model could account for migrations towards big cities; even if our model could not account for that phenomena, it offers the possibility to explore dynamics and relations with physical variables allowing SES analysis in further studies.

As far as the model development and information flow involvement was the goal of this work, no extended analysis was done over the results or scenarios. Anyhow, this model development is still descriptive and gross, is the first approach, and is specifically arranged for the variable selected. It is necessary to do future investigations as a sensitivity analysis, in order to understand the internal dynamics of the integrated physical variables or include new ones.

In the long term, the consolidation of the information production model is of interest. This requires metrics or methods for clearer quantification in various processes. Interactions between society and the ecosystem, for instance, could not be approached in this work, because the informational variable chosen is only slightly related to these fluxes. It is expected that, by better quantification of information in complex processes, the integrated model can be widely applied.

This research attempted to show how social structures represent different levels of information refinement, similar to the idea of embodied energy, which conditions the social–environment relations, and points the possibility of better characterizing information, knowledge, organization, and system successfulness.

## Figures and Tables

**Table 1:** Abbreviations of variables and mathematical expressions.

Variable	Units	$\sum_{i}^{n}p_{i}.u_{i}.\alpha_{i}=\bar{u_{t}}.A_{t}$
$A_{t}$	$\left[\frac{\mathrm{students}}{\mathrm{inhabitants}}\right]$	National training rate
$\bar{u_{t}}$	$\left[\frac{\mathrm{students}}{\mathrm{department}}\right]$	National average academic production per department.
$\alpha_{i}$	${\left[\frac{\mathrm{students}}{\mathrm{inhabitants}}\right]}_{i}$	Regional, departmental training rate
$u_{i}$	${\left[\mathrm{students}\right]}_{i}$	Number of students in a department (i).
		$\sum_{i=1}^{n}p_{i}.ce_{i}.\epsilon_{i}=\bar{ce_{t}}.E_{t}$
$E_{t}$	$\left[\frac{\mathrm{kWh}}{\mathrm{inhabitants}}\right]$	National average electrical consumption rate per capita.
$\bar{ce_{t}}.$	$\left[\frac{\mathrm{kWh}}{\mathrm{department}}\right]$	National average electrical consumption per department
$\epsilon_{i}$	${\left[\frac{\mathrm{kWh}}{\mathrm{inhabitants}}\right]}_{i}$	Regional/department per capita electrical consumption in department (i).
$ce_{i}$	${\left[kWh\right]}_{i}$	Total electrical consumption in department (i).
		$\sum_{i=1}^{n}p_{i}.cw_{i}.w_{i}=\bar{cw_{t}}.W_{t}$
$W_{t}$	$\left[\frac{m^{3}}{\mathrm{inhabitants}}\right]$	National average water demand rate per capita
$\bar{cw_{t}}$	$\left[\frac{m^{3}}{\mathrm{department}}\right]$	National average water demand rate per department
$w_{i}$	${\left[\frac{m^{3}}{\mathrm{inhabitants}}\right]}_{i}$	Regional/department per capita water demand in department (i).
$cw_{i}.$	${\left[m^{3}\right]}_{i}$	Total water demand in department/province (i).

**Table 2:** Regressive parameters for moments of the integrated model for a given year.

Moments		Equation	Correlation Coefficient
**First Moment**	=	–0.0040226 × year + 16.1782	–0.9868
**Second Moment**	=	0.0166 × year + 83.298	0.8896
**Third Moment**	=	1.87764 × year – 1638.7420	0.9987
**Fourth Moment**	=	63.91 × year – 84333.98	0.9992
**Fifth Moment**	=	1,833.981 × year – 2700238.35	0.9983
**Sixth Moment**	=	50,686.80 × year – 78541760.12	0.9969
**Seventh Moment**	=	1,406,766.07 × year – 2.249 109	0.9954
**Eighth Moment**	=	39,776,871.2 × year – 6.5099 × 10^10^	0.9939
**Ninth Moment**	=	1,149,338,351 × year – 1.9164 × 10^12^	0.9925
